# Results after primary reverse shoulder arthroplasty with and without subscapularis repair: a prospective-randomized trial

**DOI:** 10.1007/s00402-021-04024-6

**Published:** 2021-07-08

**Authors:** Nina Myline Engel, Malte Holschen, Domink Schorn, Kai-Axel Witt, Jörn Steinbeck

**Affiliations:** 1 Department of Orthopedic Surgery, University Hospital Muenster, Albert-Schweitzer-Campus 1, 48149 Muenster, Germany; 2 Present Address: Department of Orthopedic Surgery, University Hospital Essen, Hufelandstraße 55, 45147 Essen, Germany; 3 OPPK Muenster, Muenster, Germany; 4 Paracelsus-Klinik Bremen, Bremen, Germany

**Keywords:** Shoulder, Shoulder arthroplasty, rTSA, Reverse shoulder arthroplasty, Subscapularis, Prospective trial

## Abstract

**Introduction:**

Indications for reverse shoulder arthroplasties (rTSA) have increased since their development by Paul Grammont in 1985. Prosthesis design was enhanced over time, but the management of the tendon of the M. subscapularis (SSC-tendon) in primary rTSA is still a controversial subject with regard to perform a refixation or not.

**Methods:**

50 patients were randomized in a refixation group (A) and a non-refixation-group (B) of the SSC-tendon in a double-blinded fashion. SSC-function was assessed at baseline before surgery, such as 3 and 12 months after surgery. Constant–Murley-Shoulder Score (CS), American Shoulder and Elbow Surgeons Score (ASES), strength, range of motion (ROM), and pain on numeric rating scale (NRS) were measured in all examinations. An ultrasound examination of the shoulder was performed for evaluation of subscapularis tendon integrity at 3 and 12 month follow-up visits. Pain was evaluated on NRS via phone 5 days after surgery. Surgery was performed by a single experienced senior surgeon in all patients.

**Results:**

Patients with a refixation of the SSC-tendon and primary rTSA had improved internal rotation [40° (20°–60°) vs. 32° (20°–45°); *p* = 0.03] at 12 months of follow-up. Additionally, the A-group had increased CS [74 (13–90) vs. 69.5 (40–79); *p* = 0.029] 1 year after surgery. Results were strengthened by subgroup analysis of successful refixation in ultrasound examination vs. no refixation. No differences were seen in ASES and NRS 1 year after rTSA.

**Conclusion:**

SSC-tendon repair in rTSA improves CS and internal rotation 12 months after surgery.

## Introduction

Reverse total shoulder arthroplasties (rTSA) have gained increasing importance since their development by Paul Grammont in 1985. RTSAs are characterized by inverting the joint socket and head to medialize and distalize the center of rotation (COR). This modification leads to a recruitment of more deltoid fibers and an improvement of deltoid’s moment arm to achieve abduction/elevation in shoulder movement. Patients with rotator cuff arthropathy and/or pseudoparalysis [1] who have no conditions for total shoulder arthroplasties or hemiarthroplasties profit from these prostheses.

Today, the indication for rTSAs has extended. Not only in cuff tear arthropathy (CTA), but also in advanced osteoarthrosis, trauma, and rheumatism patients, rTSA is applied with good results in pain reduction and functional outcomes [2, 3]. Applications have been also extended to revision surgery after failure of prior arthroplasties and tumor surgery [4].

RTSA are applied in younger patients with good results. Meanwhile, rTSAs show similar running life as total shoulder arthroplasties (TSA) [5]. Different designs with a lateralization of the components (the COR) were developed. There are limited data that a lateralization of the COR (by lateralize humeral and/or glenoid components) might increase range of motion (ROM) and reduce complications, e.g., dislocation and scapular notching [4, 6]. Contrary to the Grammont’s design using a medialization of the COR, the new prostheses with more lateralized components, a varus humeral neck-shaft angle, and a better tension of the posterior rotator cuff might be a prevention for dislocation and scapular notching with good results and less complications [2, 7].

Nevertheless, the more lateralized rTSAs have a medialized and distalized COR in contrast to TSAs. As the dislocation rate of rTSA in the beginnings of its surgery increased, the question for the prevention of this complication was focused, looking at soft-tissue management and prosthesis design.

The subscapularis muscle is an important muscle for internal rotation and anterior stabilization in normal shoulder anatomy. The management of the subscapularis tendon (SSC) is still controversial in rTSA surgery.

Some studies recommend the refixation of the tendon as prevention for dislocation [8, 9] and to create superior internal rotation [10]. Other trials decline the refixation due to postoperative limitation of ROM, especially external rotation, and as no differences in postoperative outcomes after SSC-tendon repair were shown [11–14]. Other authors tried to address open issues by varying prostheses designs (e.g., medialization/lateralization of prostheses components and variation of the humeral neck-shaft angle) [12, 15, 16] and different suture techniques of the SSC-tendon [17, 18].

The patients subjectively evaluated outcome after surgery gains increasing importance in clinical assessment of postoperative scores. So far, there are only retrospective non-randomized studies and clear evidence is lacking for the management of the SSC-tendon in primary rTSA. The purpose of this clinical prospective, randomized trial was to close the current knowledge gap and address open questions concerning functional outcome after rTSA with or without SSC-tendon refixation.

We hypothesized that patients with SSC-tendon refixation in primary rTSA have less pain and a better internal rotation than patients without SSC-refixation. Additionally, we presumed, that patients with a more lateralized COR, a varus humeral neck-shaft angle, and a SSC-refixation demonstrate better internal rotation than patients with a Grammont’s design prosthesis and without SSC-refixation. We hypothesized that patients with a more lateralized prosthesis and a varus humeral neck-shaft angle have a better external rotation independent of SSC-refixation.

## Material and methods

The study was approved by the local ethics committee and registered in the National Clinic Trials Register.

### Study design

**Fig. 1:**
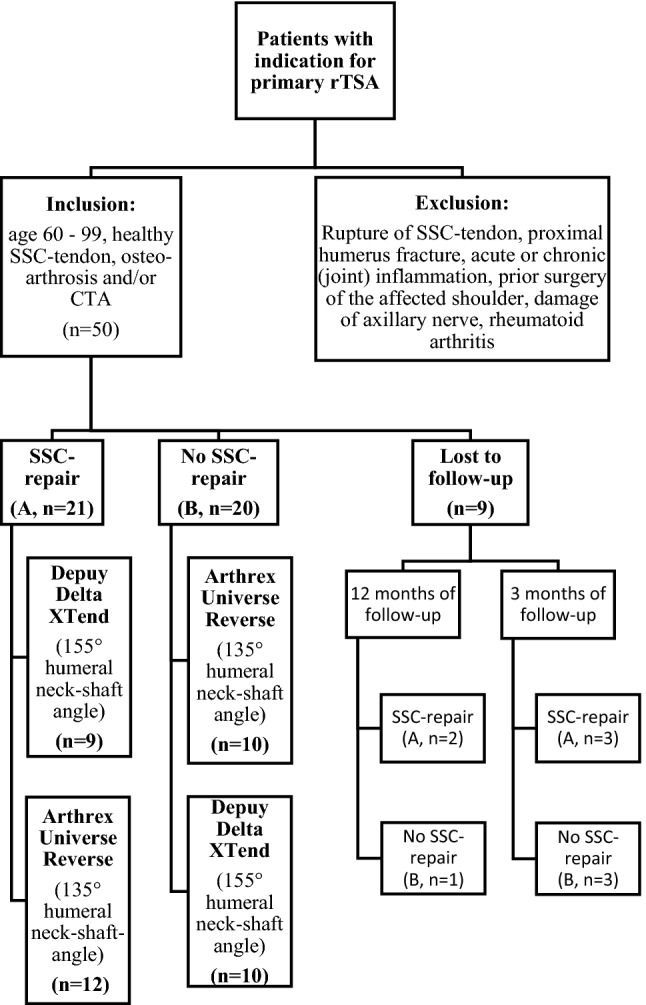
Study design with inclusion and exclusion criteria

Patients from the outpatient clinic were screened for inclusion into the study from 05/2015 to 06/2017. 50 patients undergoing primary rTSA met the inclusion criteria. They were recruited and randomized in the refixation group (A, *n* = 26) and the non-refixation group (B, *n* = 24) in a double-blinded fashion (Fig. 1). At the end of the follow-up, the data from 21 patients in group A and 20 patients in group B could be analyzed.

The randomization list was generated by a random generator in the institute for biometrics and clinical trials. Surgery was performed by one single senior surgeon. The surgeon was informed by a resident if the SSC-tendon has to be repaired or not immediately before surgery and after randomization. Patients were blinded to this decision.

Indications for surgery were CTA and osteoarthrosis. Exclusion criteria were insufficiency or tear of the subscapularis tendon, previous surgery of the affected shoulder, proximal humerus fractures, damage of the axillary nerve, and acute or chronical (joint) infection. Patients < 60 years and > 99 years were excluded as well as patients with rheumatoid arthritis. The function of the SSC-muscle was tested via clinical examination (lift-off test, belly press test, ROM) before the surgery by one skilled examiner (NE).

The primary endpoint was difference in pain on NRS after rTSA dependent on SSC management. The secondary endpoints were differences in functional outcomes as measured by CS, ASES, and ROM.

### Surgery technique

During recruitment, the type of implanted prostheses was changed as rTSA with a 135° humeral neck-shaft angle showed trends towards better results in postoperative outcome scores in other trials [6, 15, 16, 19]. 19 patients received a prosthesis with a 155° humeral neck-shaft angle and a 10° retroversion (Delta XTend Reverse; DePuy Synthese, Warsaw, Indiana, USA). Since June 2016, the Arthrex Universe Reverse prosthesis with a 135° humeral neck-shaft angle, 30° retroversion, and a + 4 mm Offset (Arthrex Universe Revers, Arthrex; Naples, Florida, USA) [20] was used in the 22 following patients.

Patients were placed in Beach-Chair-position during surgery. All patients received general anesthesia and peripheral nerve blockage. The deltopectoral approach has been used in all patients. In both groups, an L-shaped tenotomy of the SSC-tendon was performed leaving a 1-cm tendon stock on the lesser tuberosity for its later refixation. All patients had an intact subscapularis tendon. The tendon was secured in group A with polyethylene sutures. The joint capsule was dissected, a tenotomy of the long biceps tendon was performed, and the humeral head was dislocated. After preparing the bone, removing osteophytes, and preparing the soft tissue of the shoulder joint, the rTSA was implanted depending on the individual patient humeral head and glenoid size as well as manufacturer’s instructions. An individual retroversion between 10° and 30° was adjusted. After using some irrigation fluid and hemostasis, a drain was placed close to the joint.

If the patients were randomized in the A-group, the SSC-tendon was sutured with four non-absorbable orthocord sutures (DePuy Synthese, Warsaw, Indiana, USA) and a humeral site-to-site refixation in single knot technique in abduction and 20° external rotation without too much tension on the tendon. A refixation in loco typico was performed. The single soft-tissue layers were closed in a standard procedure.

### Postoperative procedure

The shoulder was immobilized for 3 weeks using an abduction sling (a sling with an abduction pillow). Three weeks after surgery, the patients started active-assisted mobilization (abduction < 60°) of the shoulder supported by a physiotherapist. Six weeks after surgery, the patients started active movement in a range free of pain to achieve a full joint mobility. Active internal and external rotation was limited in the first 6 weeks. Three months after surgery, the first follow-up took place in the outpatient clinic. If there were no contraindications, patients were allowed to increase forces on the shoulder. Most of the patients took part in a rehab program before the first follow-up in the outpatient clinic after having removed the abduction sling.

### Follow-up

The patients were seen prior to surgery, 3 and 12 months thereafter. Additionally, pain was evaluated via phone using a numeric rating scale (NRS) 5 days after surgery. An ultrasound examination to determine the integrity of the subscapularis muscle and tendon was performed 3 and 12 months after surgery. Two categories were set: intact (fibers or the complete tendon could be seen) vs. non intact SSC-tendon (no fibers could be seen). The examinations were done by the same examiner (NE).

For assessment of joint function, Constant–Murley Shoulder Score (CS) and the American Shoulder and Elbow Surgeons Score (ASES) were used. Pain was evaluated using an NRS with a range of 0–10 (10 represented the maximum of pain imaginable). Active movement in degrees was tested with a goniometer on top. Internal and external rotations were measured in abduction with forearm movement (if possible and 90° abduction could be reached). Indirect rotational movement was measured in CS looking at achievable points on the back (internal rotation) or reaching different points on/above patients head (external rotation) as described previously [21]. The strength was tested in 90° degrees flexion and 90° abduction (if possible) with a spring balance. If 90° flexion and 90° abduction could not be reached, zero points were given in the score. Adjusted CS (aCS) [22] was calculated as well.

### Statistical analysis

For statistical analysis, IBM SPSS Statistics 25 (IBM Corporation, Amonk, NY, USA) was used. Fisher’s exact test was performed to compare baseline characteristics. The Mann–Whitney *U* test, respectively, and the t test were used for comparison of two groups and for subgroup analysis. *p* values below 0.05 were considered statistically significant.

A multivariant mixed-model regression analysis with a random intercept (per person) was performed with CS in the first and internal rotation in the second model as dependent variables.

The following predictors (fixed-effects) were included: age, gender, baseline CS, treatment (refixation vs. no refixation), prosthesis (Delta Xtend vs. Univers Revers), and timepoints after surgery. The initial models were adjusted for non-significant variables to reduce disturbances. Both models underlined the influence of the refixation on CS and internal rotation.

Post hoc power analysis with G’Power 3.1.9.4 was performed using the measured means and standard deviations for CS, aCS and internal rotation 1 year after surgery (effect size *d* > 1, power > 90% for all variables).

## Results

There were no differences between the two groups regarding age, sex, handedness, CS, pain, and ASES prior to surgery. Patient characteristics at baseline are summarized in Table 1. Reasons for dropouts were an incomplete follow-up (*n* = 9; *A* = 5; *B* = 4). Reasons for the incomplete follow-up were dislocation and revision surgery in one case (group A, 155° prosthesis), one revision surgery because of components failure (group B, 135° prosthesis), and one postoperative nerve lesion (group A, 155° prosthesis). The other patients could not attend the follow-up at the outpatient clinic, due to immobility, transport, or other diseases.

**Table 1:** Baseline characteristics with preoperative functional outcome scores

	All (*n* = 41)	A (*n* = 21)	B (*n* = 20)	*p* value
Age (years) (mean and standard deviation)	72.3 (± 7.8)	71.1 (± 8.8)	73.5 (± 6.6)	0.196
Sex (male/female) (%)	29.3/70.7	38.1/61.9	20/80	0.306
Affected shoulder (right/left) (%)	63.4/36.6	61.9/38.1	65/35	1
Handedness (right/left) (%)	90.2/9.8	90.5/9.5	90/10	1
CS prior to surgery		33 (14–70)	35 (16–58)	0.473
ASES prior to surgery		32 (13–75)	26.5 (13–53)	0.187
Pain prior to surgery		6.7 (1.3–9)	6.4 (3–10)	0.823

Median and ranges for the single values are provided, *p* < 0.05 was considered statistically significant

*A* SSC-tendon repair, *B* no SSC repair, *CS* Constant–Murley Shoulder Score (points), *ASES* American Shoulder and Elbow Surgeons Score (points), pain on numeric rating scale (0–10 points)

All patients had better CS, aCS, ROM, ASES, and less pain after surgery compared to baseline parameters (*p* < 0.001). ASES did not differ between groups (refixation vs. no refixation of SSC-tendon) 3 and 12 months after surgery (Table 2). There was no difference in the primary endpoint (postoperative pain on the NRS) 3 and 12 months after surgery between both groups (Table 2). Differences between group A and B could be seen in secondary endpoints (postoperative CS, internal and external rotations, as well as postoperative abduction force 1 year after surgery). Patients with a refixation of the subscapularis tendon had a better postoperative CS than patients without the tendon repaired 1 year after surgery [74 (13–90) vs. 69.5 (40–79); *p* = 0.029, Table 2]. Besides, the patients with a repaired SSC-tendon showed better internal rotation in degrees [40° (20°–60°) vs. 32° (20°–45°); *p* = 0.03, Table 2] than the patients without the refixation. We could show an intact tendon after refixation in 16 of 21 cases (76%).

**Table 2:** Functional outcome scores and range of motions at 3 and 12 months of follow-up dependent on subscapularis refixation

	*A* *n* = 21		*B* *n* = 20		*p* value	
	3 months after surgery	12 months after surgery	3 months after surgery	12 months after surgery	3 months after surgery	12 months after surgery
CS	62 (21–76)	74 (13–90)	57 (29–82)	69.5 (40–79)	0.327	**0.029**
aCS	72 (21.2–89.7)	81.1 (13.2–96.8)	66.6 (39.8–91.5)	80.9 (42.9–88.5)	0.426	0.095
ASES	83 (18–97)	92 (8–98)	79.5 (23–95)	88 (27–98)	0.418	0.194
Pain	0 (0–6)	0 (0–8)	1.3 (0–6.7)	0 (0–7)	0.859	0.429
Strength abduction^a^	4 (1–11) *n* = 17	8 (2–25) *n* = 19	3 (2–10) *n* = 18	5 (2–11) *n* = 18	0.207	0.233
Strength flexion^a^	4 (1–13) *n* = 18	7 (2–20) *n* = 19	4 (1–10)	6 (2–14) *n* = 19	0.496	0.146
FF	124 (62–156)	140 (55–170)	119 (96–152)	137 (92–158)	0.465	0.301
ABD	114 (50–156)	130 (40–152)	113 (78–154)	123 (70–152)	0.611	0.440
IRO	40 (20–60) *n* = 20	40 (20–60) *n* = 20	40 (20–60) *n* = 19	32 (20–45)	0.531	**0.03**
IRO^a^	6 (0–10)	8 (0–10)	2 (0–10)	4 (0–10)	**0.024**	**0.025**
ARO	20 (0–40)	20 (0–35) *n* = 20	19 (0–50)	20 (0–40)	0.572	0.841
ARO^a^	8 (0–10)	10 (0–10)	10 (0–10)	10 (4–10)	0.560	**0.024**

Median and ranges for the single values are provided, *p* < 0.05 was considered statistically significant

*A* SSC-tendon repair, *B* no SSC repair, *(a)CS* (adjusted) Constant–Murley Shoulder Score (points), *ASES* American Shoulder and Elbow Surgeons Score (points), pain on numeric rating scale (0–10 points), *FF* forward flexion, *ABD* abduction, *IRO* internal rotation, *ARO* external rotation; values are given in degree

^a^Strength measured in 90° abduction and forward flexion, internal rotation (IRO) and external rotation (ARO) measured in CS, values are given as reached points in CS

Significant differences between groups are highlighted in bold

We performed further the following subgroup analysis (Tables 3, 4, 5).

**Table 3:** Functional outcome scores and range of motions at 12 month follow-up in DePuy Delta Xtend and Arthrex Universe Reverse prostheses dependent on subscapularis refixation

	DePuy Delta Xtend		*p* value	Arthrex Universe Reverse		*p* value
	A (*n* = 9)	B (*n* = 10)		A (*n* = 12)	B (*n* = 10)	
CS	75 (13–87)	69 (50–79)	0.113	72 (62–90)	71 (40–74)	0.123
aCS	83.2 (13.2–96.8)	80.9 (60.8–88.5)	0.278	80.8 (72–91.1)	79.3 (42.9–85.4)	0.159
ASES	93 (8–98)	89 (67–98)	1	92 (85–98)	87.5 (27–95)	0.14
Pain	0 (0–8)	0 (0–7)	0.243	0 (0–3)	0 (0–5.3)	0.582
Strength abduction^a^	11 (4–25)	6 (3–11)	0.016	5 (2–18)	4 (2–9)	0.862
Strength flexion^a^	10 (4–19)	6 (2–10)	0.019	5.5 (2–20)	6 (2–14)	1
FF	140 (55–148)	138 (92–158)	0.78	143 (100–170)	133 (100–154)	0.314
ABD	130 (40–140)	118.5 (80–148)	0.4	125.5 (100–152)	127.5 (70–152)	0.872
IRO	40 (30–50)	31 (25–45)	0.101	40 (20–60)	32.5 (20–40)	0.123
IRO^a^	8 (0–10)	4 (2–10)	0.447	8 (4–10)	4 (0–10)	**0.021**
ARO	18 (0–34)	7.5 (0–40)	0.573	22.5 (0–35)	24 (0–38)	0.872
ARO^a^	10 (0–10)	8 (4–10)	0.133	10 (10–10)	10 (6–10)	0.722

Median and ranges for the single values are provided, *p* < 0.05 was considered statistically significant

*A* SSC-tendon repair, *B* no SSC repair, *(a)CS* (adjusted) Constant–Murley Shoulder Score (points), *ASES* American Shoulder and Elbow Surgeons Score (points), pain on numeric rating scale (0–10 points), *FF* forward flexion, *ABD* abduction, *IRO* internal rotation, *ARO* external rotation; values are given in degree

^a^Strength measured in 90° abduction and forward flexion, internal rotation (IRO) and external rotation (ARO) measured in CS, values are given as reached points in CS

Significant differences between groups are highlighted in bold

**Table 4:** Functional outcome scores and range of motions at twelve months follow-up in DePuy Delta Xtend and Arthrex Universe Reverse prostheses independent of subscapularis refixation

	Depuy Delta Xtend (*n* = 19)	Arthrex Universe Reverse (*n* = 22)	*p* value
CS	71 (13–87)	71 (40–90)	0.875
aCS	80.9 (13.2–96.8)	80.8 (42.9–91.1)	0.875
ASES	90 (8–98)	91 (27–98)	0.733
Pain	0 (0–8)	0 (0–5.3)	0.887
Strength abduction^a^	7 (3–25)	4 (2–18)	**0.044**
Strength flexion^a^	7 (2–19)	6 (2–20)	0.322
FF	138 (55–158)	140 (100–170)	0.3
ABD	125 (40–148)	127.5 (70–152)	0.276
IRO	39 (25–50)	38 (20–60)	0.545
IRO^a^	8 (0–10)	6 (0–10)	0.873
ARO	14 (0–40)	22.5 (0–38)	0.338
ARO^a^	10 (0–10)	10 (6–10)	**0.012**

Median and ranges for the single values are provided, *p* < 0.05 was considered statistically significant

*(a)CS* (adjusted) Constant–Murley Shoulder Score (points), *ASES* American Shoulder and Elbow Surgeons Score (points), pain on numeric rating scale (0–10 points), *FF* forward flexion, *ABD* abduction, *IRO* internal rotation, *ARO* external rotation; values are given in degree

^a^Strength measured in 90° abduction and forward flexion, internal rotation (IRO) and external rotation (ARO) measured in CS, values are given as reached points in CS

Significant differences between groups are highlighted in bold

**Table 5:** Constant Score, adjusted Constant Score, and internal rotation values at 12 month follow-up in patients with successful refixation and no refixation

	Successful refixation (*n* = 16)	No refixation (*n* = 20)	*p* value
CS	76.13 (± 7.446)	65.70 (± 10.327)	**0.002**
aCS	85.10 (± 7.014)	74.98 (± 11.562)	**0.004**
IRO	43.47 (± 8.079) (*n*=15)	33.15 (± 7.485)	**0.001**
IRO^a^	7.88b(± 2.125)	4.70 (± 3.326)	**0.001**

Mean and standard deviations for the single values are provided, *p* < 0.05 was considered statistically significant

*(a)CS* (adjusted) Constant–Murley Shoulder Score (points), *IRO* internal rotation, values are given in degree

^a^Internal rotation (IRO) measured in CS, values are given as reached points in CS

Significant differences between groups are highlighted in bold

### SSC-refixation vs. no refixation in DePuy Delta XTend prostheses

Patients with a 155° humeral neck-shaft angle prosthesis and a repaired SSC-tendon had a better force tested in reachable points in CS in 90° abduction [11 (4–25) vs. 6 (3–11); *p* = 0.016, Table 3], compared to the non-repair group 1 year after surgery.

### SSC-refixation vs. no refixation in Arthrex Universe Reverse prostheses

In contrast, using a 135° humeral neck-shaft angle prosthesis under the aspect of SSC management, patients with an SSC-refixation showed better internal rotation in reachable points in CS 1 year after surgery (8 (4–10) vs. 4 (0–10); *p* = 0.021, Table 3), compared to the patients without a repair.

### Successful refixation versus no refixation

Group A with successful refixation in the ultrasound examination (*n* = 16) showed a significant difference compared to group B (*n* = 20) for CS [76.13 (± 7.446) vs. 65.70 (± 10.327), *p* = 0.002] and aCS [85.10 (± 7.014) vs. 74.98 (± 11.562), *p* = 0.004] (Table 5).

Furthermore, a benefit in the successful refixation group could be demonstrated for internal rotation in degrees [43.47 (± 8.079) vs. 33.15 (± 7.485), *p* = 0.001] and points in CS [7.88b (± 2.125) vs. 4.70 (± 3.326), *p* = 0.002] compared to the entire group B (Table 5).

### Comparison of functional outcomes from different prostheses independent from SSC management

Patients who underwent surgery with a 135° neck-shaft angle prosthesis (Arthrex Universe Reverse) showed better external rotation measured in reachable points in CS 1 year after surgery [10 (6–10) vs. 10 (0–10); *p* = 0.012, Table 4], compared to those that received a 155° neck-shaft angle prosthesis (DePuy Delta XTend) independent of SSC repair. Patients with a 155° neck-shaft angle prosthesis had a better postoperative force measured in 90° abduction in points of CS [7 (3–25) vs. 4 (2–18); *p* = 0.044, Table 4], compared to patients receiving a 135° neck-shaft angle prosthesis independent of SSC repair.

## Discussion

Since the development of rTSAs, the management of the SCC-tendon, if it is repairable, has been discussed controversially as no concise data exist so far. To our knowledge, this is the first prospective-randomized trial analyzing the management of the SSC-tendon in primary rTSA. Only retrospective or non-randomized studies existed prior to our study [6, 10, 11, 13, 14, 23, 24]. Malahias et al. [23] did a review of the most common (retrospective) studies on this question [6, 10, 11, 13, 14], focusing on functional outcomes and dislocation rates after rTSA. They concluded that a reattachment of the SSC-tendon does not lead to a clinical benefit as only Friedmann et al. [10] could show increased CS and internal rotation in the refixation group. The other studies [6, 11, 13, 14] analyzed in the review could not confirm these findings. On the contrary, Matthewson et al*.* [24] focused on the dislocation rate of rTSA in their meta-analysis depending on SSC-reattachment and prostheses design (medialization and lateralization of the COR) and concluded that SSC-tendon repair in medialized rTSA (Grammont’s design) decreases the risk of dislocation. A recommendation to use a more lateralized rTSA-design when the SSC-tendon is not repairable was given to reduce the risk of dislocations [24].

Concerning functional outcomes, our study is in line with other studies [10–12] demonstrating a benefit of rTSA surgery independent from SSC management. A limitation of prior studies was availability of comprehensive functional assessment scores of shoulder function, and only single parameters were assessed leaving open questions in current patient care. CS was only measured by De Boer et al. [13]., Vourazeris et al. [14], and Friedman et al. [10], while Werner et al.[6] focused on ASES and Clark et al.[11] only mentioned pain on NRS and ROM.

We tried to fill this gap by measuring a variety of established parameters for assessing shoulder function after primary rTSA, including CS and adjusted CS which are established scores in shoulder examination [25, 26].

A further strength of our study is that one single experienced senior surgeon performed surgery in all patients and that one examiner did the examination prior to and after surgery in all patients.

In only about 50% of the major studies on this topic, the surgery was performed by a single surgeon [12–14]. As it is a proven fact that functional outcomes after surgery are dependent on the surgeon’s experience and learning curve, as well as intraoperative decisions concerning prosthesis design, soft-tissue management, and rehab program after surgery [27–29].

The SSC-tendon has been refixated in other studies [6, 8, 10, 11, 13, 14] depending on the intraoperative quality of the muscle and the tendon, the tension-free possibility to repair it, or the surgeon’s individual decision. A strength of our study was the definition of strict inclusion criteria, leading only to the inclusion of patients with intact SSC-tendon as diagnosed clinically prior to surgery, no previous surgeries, and a randomization of patients prior to surgery.

Strength as well as weakness of our study is the evaluation of the SSC after surgery. Most of the studies which evaluated the SSC management have not examined the tendon and muscle integrity in the follow-up [6, 10–12, 14]. It would be more sensitive and valid if pre- and postoperative MRI (with metal artifact reduction) had be done as the results of ultrasound examination depend on examiner abilities [30, 31]. However, we could not find structured imaging before and after surgery in any trial.

We could show an intact tendon and muscle after refixation in 16 from 21 cases (76%). These rate is higher than described by de Boer et al. [13] (40% SSC-sufficiency in outpatient clinic control). When comparing the successful refixation group versus the no refixation group, we found a mean difference in CS from 10.43 points. According to Simovitch et al. [32], a difference above 5.7 points in the CS is the minimal clinically important difference (MCID) and clinical benefits for the patient are obtained. A significant difference for aCS could be shown in the subgroup analysis, as well. Furthermore, a difference from 3.18 points for internal rotation in CS could be demonstrated which is above the MCID (> 2) reported from Torrens et al. [33]. Thus, our study clearly shows benefits of subscapularis refixation for internal rotation and CS. The additionally performed mixed-model regression analysis and post hoc power analysis underlined the results as not incidental findings.

It must be pointed out that the validity of these results is limited by initial muscle atrophy, abilities of the examiner, the patient’s testing conditions, and metal artifacts in ultrasound examinations after shoulder arthroplasties [34].

Unlike Hanse et al. [35] who considered that SSC repair in rTSA decreases functional outcomes like external rotation and prostheses lifetime, we could not confirm these findings.

In contrast to the results of Franceschetti et al. [12], Vourazeris et al. [14], Werner et al. [6], and Clark et al. [11], we could show significantly improved internal rotation as well as CS after surgery in the SSC repair group over all prostheses designs. Our results are in line with the data from Friedman et al. [10].

In subgroup analyses, we could figure out significantly increased internal rotation in the SSC-refixation group in 135° humeral neck-shaft angle prostheses and a more lateralized COR 1 year after surgery compared to the non-repair group with the similar prosthesis design. This is also in line with the results of a retrospective trial from Friedman et al*.* [10] that demonstrated that SSC repair in prostheses design with a similar humeral neck-shaft angle lead to significant increases in internal rotation. These results were also demonstrated by Dedy et al. [36]*.*

We could confirm the functional improvement [16] in a more lateralized COR and a lower humeral neck-shaft angle as our subgroup analysis (155° humeral neck-shaft angle vs. a 135° humeral neck-shaft angle) demonstrated a significant increase of external rotation in the Arthrex Universe Reverse prosthesis.

## Limitations

A major weakness of our study is the small number of patients included, as the recruitment for primary rTSA is getting more difficult because of strict inclusion criteria, previous shoulder surgery in patients, or SSC-insufficiency. This issue was further aggravated by switch of prosthesis designs due to recent data [16]. A future prospective-randomized study on primary rTSA should include patients being operated by the same surgeon using different prostheses designs (medial glenosphere and lateral humerus, medial humerus and lateral glenosphere, and both components lateral) with and without SSC repair.

As Friedman et al. [37], Monir et al. [38], and Leathers et al. [39] demonstrated an increase in frequency of rTSA in younger patients, an extension of inclusion criteria seems reasonable for further studies, especially as younger patients tend to be in better physical condition prior to surgery with a better capacity for improvement after surgery [37, 40–42].

X-rays and/or MRI should be included regularly in future trials.

As we used the deltopectoral approach in all patients, it may be helpful to compare the different approaches regarding postoperative SSC-function and general outcomes like Lädermann et al. [43] did. Recovery time after surgery (and therefore former begin of rehab program) seemed to be superior using a SSC-preserving approach [43, 44].

As we only used one type of refixation technique of SSC-tendon, it could be interesting to do further research on different sutures techniques [18] in different prostheses designs.

A focus on pre- and postoperative physical abilities and patients expectations [45, 46], as well as an individual rehab program could be helpful in future studies to range patients outcomes and to find the best treatment option. Carbacas et al. [47] could show rapid functional improvement 1 year after surgery in subjective patient-reported outcomes measures (PROMs) and not thereafter. Questionnaires including PROMs and rate of return to sports activity [40] should be additionally used in future studies to interpretate statistically significant findings in the context of clinical relevant results and patients expectations to create a more patient-centered medicine.

## Conclusion

There is a huge mass of only retrospective studies concerning the management of SSC-tendon in rTSA. The results of our prospective-randomized study indicate that patients with SSC-refixation and primary rTSA have better results in postoperative CS and internal rotation in short time follow-up. Larger prospective-randomized trials and a longer follow-up including questionnaires to assess subjective reported outcomes are necessary to determine the long-term outcome of different designs of rTSA with SSC repair.
